# Analyses of Grains and Vegetables, Distinguishing the Nitrogenous from the Non-nitrogenous Ingredients, for the Purpose of Estimating Their Separate Values for Nutrition

**Published:** 1847-03

**Authors:** 


### BIBLIOGRAPHICAL NOTICES.

ART. VII.—*Analyses of Grains and Vegetables, distinguishing the Nitrogenous from the Non-nitrogenous Ingredients, for the Purpose of estimating their separate Values for Nutrition. Also, on Ammonia found in Glaciers; and on the Action and Ingredients of Manures.* By E. N. HORSFORD, A. M. Boston, James Munroe and Company, 1846: pp. 68.

One of the most important facts in vegetable physiology is the observation made by Bonnet, that plants growing in rain or river water give out bubbles of air. The same observer remarked, that when plants grew in water which had been boiled, their growth was not attended with the evolution of gas. Priestley took up the subject, and, in that spirit of research for which he was distinguished above all his cotemporaries, applied to the gas such tests as showed it to be oxygen. Another valuable observation added by Ingenhousz was, that it was only when growing in the sun that plants had the power to extricate this gas. It was reserved for a later philosopher to show whence the oxygen was derived, and this was done by Sennebier, who proved that it came from carbonic acid. Finally, Percival and Saussure demonstrated that plants grew more rapidly in an atmosphere containing an excess of this acid. Boussingault has recently established that one part of carbonic acid in eleven of atmospheric air is highly favorable to the growth of vegetables so long as they are under the influence of the solar rays, but that in the shade the smallest quantity of the gas is injurious to them. Their growth was found to be hastened by fanning them with an air loaded with the acid, and the air which had been subjected to their action proved to be strained of the gas. Hence it results that carbonic acid is the food of plants.

It is only within a few years that the extent and importancce of nitrogen in plants has come to be recognized. Not very long ago chemists regarded this as constituting the great distinction between vegetable and animal matters, that the former were nearly destitute of nitrogen, whilst in the latter it was an abundant element. Gay-Lassac was the first to show that nitrogen is an almost invariable constituent in the seeds of plants, and since this important discovery the conception of animal nutrition has been assuming a character more and more definite, until what was regarded as a most mysterious process has come to appear a very simple one. Modern chemical research has disclosed the interesting fact, that vegetable substances may be arranged in two classes, the nitrogenized and the non-nitrogenized. Animals die of inanition if confined to a diet destitute of nitrogen, and laborers supplied only with food containing none of this principle become incapable of executing their tasks. This is all easily explained, and depends upon the identity in chemical composition between vegetable fibrin, albumen and caseine, and the corresponding bodies found in the animal kingdom. The aliment must contain matters suited to replace the animal tissues consumed. This being understood, the chemist, when he has ascertained the relative proportion of the nitrogenous compounds in various kinds of food, is prepared to say what is their relative value. The more nitrogen, the more nourishment there is in them.

"The nitrogenous bodies, from their solution in blood, form the tissues—the actual organism. The bodies wanting nitrogen contribute, by their more or less perfect combustion, to the warmth of the animal body; and the salts of the alkalies and alkaline earths, serve in building up the osseous framework, beside constituting an essential part of every organ of the animal system.

Their values for the latter purpose are in proportion to the phosphates the ashes contain.

Their values for the second purpose above mentioned, may be considered, in general, as in the inverted relation of their values for the the first;—since the larger the proportion of nitrogenous bodies, the less must be the proportion of bodies wanting nitrogen.

Their values for the first purpose, that of ministering to the support and growth of organic tissues, have been the specific object of the hereafter enumerated determinations."

The object of the investigation the results of which are detailed in these Essays, was to determine the relative values of different kinds of vegetable food, and was undertaken at the suggestion, as the author states, and under the direction of Professor Von Liebig, in the Giessen Laboratory. It has been pursued to a highly successful and creditable termination. The author shows himself capable of the highest achievments in analytical chemistry, and we look upon his present effort as but an earnest of the contributions which may be expected from him to this most interesting department of science. It is only his results which would be interesting to most readers, and these are presented in the following table, the last column of which exhibits the conclusions of Boussingault formed upon experiments with a view to practical equivalents. It may, perhaps, be well enough to explain, that the smallest numbers express the highest values. Thus, 100 pounds of wheat are equivalent to 1000 pounds of turnips, and 55.5 pounds of linsen to 100 pounds of wheat.

*Tabular View of Nutriment Values expressed in equivalents, Wheat at* 100.

| Name. | Theory. | | Experiment |
|---|---|---|---|
| | Dried at 100° C. | In fresh condition. | In fresh condition. |
| Wheat | 100. | 100. | 94. |
| Rye | 98.8 | 97.6 | 97.6 |
| Indian Corn | 115. | 113. | 108. |
| Triticum Monococcum | 128. | 124.6 | —— |
| Barley | 104. | 102. | 101.5 |
| Panicled Oats | 92. | 90. | 112.7 |
| The same, without chaff | 78. | 76.3 | —— |
| Kamschatka Oats | 110. | 106. | 112.7 |
| Common Rice | 220. | 225. | —— |
| Tartarian Buckwheat | 170. | 166. | 122.7 |
| Table Peas | 59.9 | 57.6 | 90.7 |
| Field Peas | 57.7 | 60. | 90.7 |
| Table Beans | 59.2 | 57. | 90.7 |
| Large White Beans | 58.8 | 57. | 94.7 |
| Linsen | 55.5 | 53. | —— |
| White Potatoes | 169.8 | 565.6 | 429. |
| Blue Potatoes | 220.8 | 596.3 | 429. |
| Carrots | 158.6 | 959.4 | 545.4 |
| Red Beets | 109. | 501.5 | —— |
| Yellow French Beet | 146. | 689.5 | 643. |
| Ruta Baga | 182.7 | 919.4 | 589.7 |
| White Turnips | 133.8 | 919.4 | 1000. |
| Onions | 224.6 | 210.6 | —— |

The author, as the result of his investigations, arrives at the following conclusions:

"That the same species of cereal grain, grown on different soils, may yield unequal percentages of nitrogen.

That wheat and rye flours, to the eye and sense of feeling undistinguishable from each other, may differ by from one to three tenths of their whole quantity of nitrogen.

That one seventh of fresh, ripe, cereal grains, is moisture, and may be expelled at a temperature of 100° C.

That root crops, grown on different soils, may yield unequal percentages of nitrogen.

That the percentage of moisture in edible roots, is a constant quantity for each variety.

That beets, carrots and turnips have a larger percentage of moisture than potatoes.

That more aliment is contained in a given weight of peas, beans or lentils, than in an equal weight of any other kind of food above analyzed.

That in several of the grains and roots analyzed, there are organic bodies beside those identical in composition with gluten and starch.

That the ashes of carrots. beets, turnips and potatoes, as Prof. v. Liebig has already remarked, contain carbonates.

That the ashes of all the varieties of vegetable food above analyzed contain iron.

Finally, that the difference between the theoretical equivalents of vegetable food, as estimated from the percentage of nitrogen, and those ascertained by the experiments of stock growers, and the differences between the results of different stock growers, may be attributed in part to the unequal percentages of nitrogen in the grains and roots of the same species or variety, when grown on different soils,—but chiefly to the imperfection of determining the modes of the practical equivalents. These have been imperfect,

1st. Because the prominent test employed, has been increase or diminution in weight, of the animal fed. Increase in weight may arise from secretion of fat derived from the sugar and starch of plants. Diminution in weight may follow unusual activity, increasing the consumption of fat already present.

2d. Because theoretical equivalents have been employed in conditions unequally suited to digestion. The same article of food, coarse or fine, fresh or prepared for easy digestion, yields unequal measures of nutrition.

3d. Because the experiments, in but few instances, have been made with substances where moisture and nitrogen had been previously ascertained."

Mr. Horsford analysed the water obtained from a glacier of the Alps, and ascertained that it contained two parts of ammonia in the million; from which it is evident that ammonia is not confined to the lower strata of the air. Mr. H. also infers,

"That a shower of rain, after a long interval of fair weather, should produce, through the ammonia descending with it, immediate effect in vegetation; and

That a soil, containing the necessary inorganic matters, and whose physical properties enable it to retain a certain measure of moisture, should be fruitful, inasmuch as it retains the ammonia with the moisture; and

That a soil containing so large a proportion of clay that it does not permit water to filter through, should be less fruitful, since the subsequent falls of rain, after the soil has become filled, will flow away, and with them the ammonia they have brought down."

Ammonia exists in the soil in very surprising quantities, as appears from the annexed table, for which we are indebted to the work before us:

| Name of Soil Examined. | Ammonia in a stratum one acre in area, and one foot deep, in pounds, avoir. |
|---|---|
| Clay soil, before manuring | 9751 |
| Clay soil | 9463 |
| Surface soil, from Hohenheim | 8985 |
| Subsoil, from the same field | 6015 |
| Clay soil, before manuring | 8617 |
| Clay soil " " | 8502 |
| Soil for Barley | 8373 |
| Clay soil, before manuring | 8039 |
| Loam | 7939 |
| Loam | 7820 |
| Illinois prairie soil | 6069 |

Upon which the author remarks,

"Now, what farmer ever carted from his manure yards 8000 pounds of ammonia to an acre of land? One may almost inquire, what farmer ever carted the tenth, or even the twentieth part of this amount? It is obvious that the ammonia spread on fields in the ordinary distribution of barn yard products, is of no moment. The quantity which usually falls of rain, greatly exceeding in the course of a season, any conceivable supply of human instrumentality. These results put the question of the source of ammonia or of nitrogen out of all doubt."

If, then, ammonia is supplied in sufficient quantity from the atmosphere, to what are we to attribute the efficacy of stable manures? This author thinks, with Liebig, that the good effects are due to the alkalies and earths which they afford; and he concludes that the principle of rational improvement of soils is,

"1st. *A proper physical constitution* for the retention of moisture, escape of surplus rains, expansion of roots, etc. This will be derived from the plow, harrow, spade and hoe, and admixtures of sand in some soils, clay in others, loam in others, and organic refuse in most.

2d. A supply of the inorganic ingredients which the ashes of the plant to be cultivated contains, in such a state that they may be taken readily into the vegetable system, and yet not so soluble as to be washed away by rains."

This view of the subject certainly has simplicity, if nothing else, to recommend it. The air furnishes ammonia and carbonic acid, the great articles of vegetable nutrition; the farmer must see that the soil is fitted to receive them, and also that it contains the requisite amount of the inorganic matters by which plants are developed, and all is done that is required for plentiful crops. The rains effect the rest.

We commend heartily this elaborate tract to the perusal of all who feel an interest in the culture of the soil, of which our conception is that of the Roman orator: "Omnium rerum quibus aliquid exquiritur, nihil est agricultura melius, nihil uberius, nihil dulcius, nihil homine libero dignius."

